# The orphan nuclear receptor small heterodimer partner is required for thiazolidinedione effects in leptin-deficient mice

**DOI:** 10.1186/s12929-015-0133-3

**Published:** 2015-05-08

**Authors:** Hsiu-Ting Tseng, Young Joo Park, Yoon Kwang Lee, David D Moore

**Affiliations:** Department of Molecular and Cellular Biology, Baylor College of Medicine, One Baylor Plaza, Houston, Texas USA; 300 Gumi-dong, Bundang-gu, Department of Internal Medicine, Seoul National University Bundang Hospital, Seongnam-si, Kyeonggi-do South Korea; Department of Integrative Medical Sciences, Northeast Ohio Medical University, Rootstown, OH USA

**Keywords:** SHP, Troglitazone, PPARγ, Thiazolidinedione

## Abstract

**Background:**

Small heterodimer partner (SHP, NR0B2) is involved in diverse metabolic pathways, including hepatic bile acid, lipid and glucose homeostasis, and has been implicated in effects on the peroxisome proliferator-activated receptor γ (PPARγ), a master regulator of adipogenesis and the receptor for antidiabetic drugs thiazolidinediones (TZDs). In this study, we aim to investigate the role of SHP in TZD response by comparing TZD-treated leptin-deficient (*ob/ob*) and leptin-, SHP-deficient (*ob/ob*;*Shp*^−/−^) double mutant mice.

**Results:**

Both *ob/ob* and double mutant *ob/ob*;*Shp*^−/−^ mice developed hyperglycemia, insulin resistance, and hyperlipidemia, but hepatic fat accumulation was decreased in the double mutant *ob/ob*;*Shp*^−/−^ mice. PPARγ2 mRNA levels were markedly lower in *ob/ob*;*Shp*^−/−^ liver and decreased to a lesser extent in adipose tissue. The TZD troglitazone did not reduce glucose or circulating triglyceride levels in *ob/ob*;*Shp*^−/−^ mice. Expression of the adipocytokines, such as adiponectin and resistin, was not stimulated by troglitazone treatment. Expression of hepatic lipogenic genes was also reduced in *ob/ob*;*Shp*^−/−^ mice. Moreover, overexpression of SHP by adenovirus infection increased PPARγ2 mRNA levels in mouse primary hepatocytes.

**Conclusions:**

Our results suggest that SHP is required for both antidiabetic and hypolipidemic effects of TZDs in *ob/ob* mice through regulation of PPARγ expression.

## Background

Obesity is associated with cardiovascular disease, type 2 diabetes mellitus and some cancers [1,2]. Among these, type 2 diabetes mellitus is a major source of mortality in the obese population. Diabetes develops in the context of both insulin resistance and β- cell dysfunction [3]. In insulin resistance, the ability of insulin to enhance glucose disposal in muscle and adipose tissue and to decrease gluconeogenesis in liver is impaired. Diabetes ensues when the pancreatic β-cell cannot compensate for insulin resistance by adequately increasing insulin secretion.

Thiazolidinediones (TZDs) are a class of antidiabetic drugs that act by increasing insulin sensitivity [4,5]. TZDs, including troglitazone, rosiglitazone and pioglitazone, improve insulin action in patients and a number of insulin-resistant obese and diabetic murine models, such as *ob/ob* (*leptin*-deficient), *db/db* (*leptin receptor*-deficient), KKA^y^ mice and Zucker fatty rats [6-8]. TZDs are potent agonist ligands for the nuclear hormone receptor peroxisome proliferator-activated receptor γ (PPARγ) and their antidiabetic actions are believed to be mediated by interactions with PPARγ [9,10]. PPARγ is a key regulator of adipogenesis [11,12] that exists as two protein isoforms, PPARγ1 and γ2, arising from differential promoter usage. PPARγ2 encodes 30 additional amino acids at the N-terminus compared to PPARγ1. PPARγ2 is expressed at highest levels in adipose tissue compared to other major insulin target tissues, such as liver and muscle, whereas PPARγ1 is expressed at relatively low levels in many tissues [11,13]. The expression pattern suggests that adipose tissue is the primary target of TZD-induced insulin sensitization as generally supported by tissue-specific PPARγ knockout studies, although other tissues and cell types also contribute [14-18]. PPARγ expression levels can change under different physiological conditions and affect the response to TZD treatment [13]. For example, hepatic PPARγ expression is elevated in animals that develop fatty livers [18-20], and increased PPARγ2 expression is correlated with increased liver fat in human subjects with non-alcoholic fatty liver disease (NAFLD) [21]. TZD effects could be amplified in such PPARγ-rich fatty livers, which may be particularly relevant for the beneficial effects of TZD treatment in human patients with non-alcoholic steatohepatitis (NASH) [22,23].

Mutations in the small heterodimer partner (SHP, NR0B2) have been associated with mild obesity in several human populations [24-27]. SHP is an atypical orphan nuclear receptor that lacks a conventional DNA-binding domain [28,29]. Although SHP interacts with several nuclear receptors by acting as a repressor [28,30-32], it has been reported to increase the transcriptional activation of PPARγ [33]. In addition, hepatic PPARγ gene expression is upregulated in transgenic mice expressing SHP in the liver, suggesting that SHP may affect PPARγ expression at the transcription level [34].

To investigate the role of SHP in TZD response in obese diabetic mice, we compared glucose metabolism and lipid profiles in *ob/ob* and *ob/ob*;*Shp*^−/−^ double mutant mice after TZD treatment. Troglitazone did not reduce glucose or circulating triglyceride levels in the *ob/ob*;*Shp*^−/−^ mice, which showed markedly decreased PPARγ expression in liver and, to a lesser extent, adipose tissue. Furthermore, SHP overexpression increased PPARγ mRNA levels in primary hepatocytes. These results suggest that SHP is required for TZD effects in *ob/ob* mice and for a potential indirect activation of PPARγ gene.

## Methods

### Animals and treatments

*Shp*^−/−^ mice were generated previously in this laboratory with a mixed C57BL/129sv hybrid background [35]. They were backcrossed to C57BL/6 J mice for 10 generations to obtain >99.99% pure C57BL/6 J background. The *leptin*-deficient *ob/ob* and *ob/ob*;*Shp*^−/−^ mice were generated as described previously [36]. Groups of 10–15 male *ob/ob* and *ob/ob*;*Shp*^−/−^ mice (7- to 8-week-old) were oral gavaged with vehicle (10% dimethylsulfoxide (DMSO) in corn oil) or troglitazone (Cayman chemical, dissolved in DMSO, 10 mg/kg/day) for two weeks. Before the first day of treatment and on the day before sacrifice, mice were fasted overnight and blood samples were collected from the orbital plexus. Livers and white adipose tissue were dissected, weighed and fixed for histological analysis, or snap frozen in liquid nitrogen and stored at −80°C until use. Mice were maintained in the accredited pathogen-free facility at Baylor College of Medicine on a 12-hour light/dark cycle and fed a standard rodent chow and water *ad libitum*. All protocols for animal use were approved by the animal care committee of Baylor College of Medicine.

### Histological analysis

Livers were fixed, dehydrated and embedded in paraffin. Sections were cut with a thickness of 5 μm and stained with Harris hematoxylin-eosin (Sigma).

### Serum and tissue chemistry

Serum was prepared from whole blood and stored at −80°C until use. Lipids were extracted from liver using chloroform-methanol extraction [37]. Enzymatic assay kits were used for the determination of non-esterified fatty acids (Wako), cholesterol and triglyceride (Thermo Electron). Insulin levels were measured by a mouse/rat insulin ELISA kit (Millipore-Linco).

### Glucose tolerance test

Glucose tolerance tests (GTT) were performed by intraperitoneal injection of glucose (2 g/kg of body weight) following overnight fasting. Blood samples were taken at 0, 15, 30, 60, 120 minutes from the tail vein and were analyzed for glucose concentrations using kits from Thermo Electron.

### RNA isolation and real-time quantitative PCR

Total RNA was isolated using TRIzol reagent (Invitrogen). 1 μg of total RNA was reverse transcribed using QuantiTect Reverse Transcription kit (Qiagen) according to manufacturer’s instructions. Real-time quantitative PCR (SYBR green) analysis was performed on an ABI prism 7700 sequence detection system (Applied Biosystems) under factory default thermal cycling conditions (50°C, 2 min; 95°C, 10 min; and 40 cycles at 95°C, 15 s; 60°C, 1 min). Expression was normalized to 36B4 and the relative quantification was calculated using ΔΔCt formula.

### Primary hepatocyte isolation, culture and adenoviral transduction

Primary hepatocytes were prepared from 8- to 12-week-old wild type mice by *in situ* perfusion and single-step Percoll gradient centrifugation [35]. Cells were plated at 10^6^ per 6-cm dish and grown in Williams’ E medium supplemented with 10 μg/ml transferrin, 10 μg/ml insulin, 100U/ml penicillin and 100 μg/ml streptomycin. One day after plating, the cells were infected with a SHP-expressing adenovirus or a control virus expressing GFP as described [38] for two hours at a multiplicity of infection (MOI) of 20. Virus-containing media were removed and cells were cultured for two days after infection. Total RNA were isolated from cells for real-time quantitative PCR analysis.

### Statistical analysis

Values are presented as means ± SEM. Statistical significance was determined by two-tailed *t* test or ANOVA, as appropriate.

## Results

### Troglitazone does not improve the diabetic syndromes in *ob/ob*;*Shp*^−/−^ mice

**Figure 1:**
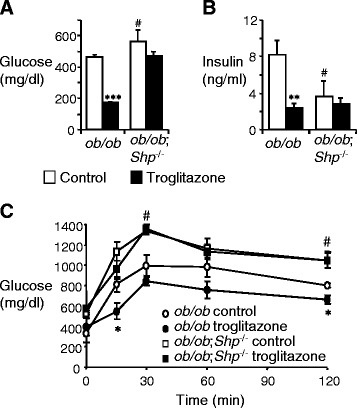
SHP deficiency causes non-responsiveness to antidiabetic effect of TZDs. **(A, B)** Serum glucose **(A)** and insulin **(B)** levels under fasting conditions. 7–8 week-old male *ob/ob* and *ob/ob*;*Shp* ^−/−^ mice were treated with control (open bars) or troglitazone (filled bars) for 2 weeks. **(C)** Glucose tolerance tests. Intraperitoneal glucose tolerance tests were performed on *ob/ob* and *ob/ob*;*Shp* ^−/−^ mice treated with control (open symbols) or troglitazone (filled symbols) for 2 weeks. n = 4–5 per group. Data are mean ± SEM. *P < 0.05, **P < 0.01, ***P < 0.001 for differences between control and troglitazone-treated *ob/ob* mice. #P < 0.01 for differences between *ob/ob*;*Shp* ^−/−^ (with no effect of troglitazone treatment) and control-treated *ob/ob* mice.

The *ob/ob* mouse is a valuable type 2 diabetes model. Based on the role of the orphan nuclear receptor SHP in metabolic pathways, we generated *ob/ob*;*Shp*^−/−^ double mutant mice. The obesity of the double mutants was not different from the *ob/ob* mice (9-10-week-old *ob/ob*;*Shp*^−/−^ body weight 38.38 ± 1.9 g versus *ob/ob* 35.8 ± 1.7 g). We initially assessed the effects of SHP deficiency on glucose homeostasis by measuring blood glucose and insulin levels. Glucose levels of *ob/ob*;*Shp*^−/−^ mice were significantly higher than those of *ob/ob* mice, whereas the insulin level was markedly lower (Figure 1A, B). To further characterize glucose metabolism, glucose tolerance tests were performed and *ob/ob*;*Shp*^−/−^ mice were more glucose-intolerant compared to *ob/ob* mice (Figure 1C). These results suggest that SHP deficiency aggravates hyperglycemia and insulin resistance in *ob/ob* mice, which is quite different from the improvements previously described [36]. The basis for this marked discrepancy is not clear.

To test whether TZDs are effective in *ob/ob*;*Shp*^−/−^ mice with severe glucose intolerance, both *ob/ob* and *ob/ob*;*Shp*^−/−^ mice were treated with troglitazone for 2 weeks. As expected, the *ob/ob* mice showed dramatically lower serum glucose and insulin levels, as well as improved glucose tolerance (Figure 1). In contrast, neither the serum glucose and insulin levels nor the glucose tolerance was improved in the *ob/ob*;*Shp*^−/−^ mice. These results indicate that SHP is required for antidiabetic effects of TZDs in *ob/ob* mice.

### Troglitazone has no effect on the lipid profile of *ob/ob*;*Shp*^−/−^ mice

**Figure 2:**
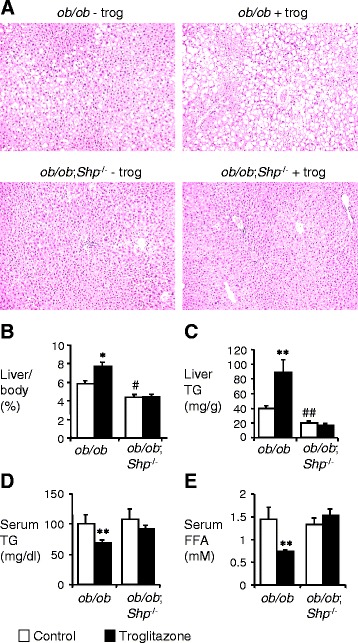
SHP deficiency blunts TZD effects on lipid profile of *ob/ob* mice. **(A)** Histology of livers from *ob/ob* and *ob/ob*;*Shp* ^−/−^ mice treated with control or troglitazone for 2 weeks. H&E staining was performed for liver sections. ×100 magnification. **(B-E)** 7–8 week-old male *ob/ob* and *ob/ob*;*Shp* ^−/−^ mice were treated with control (open bars) or troglitazone (filled bars) for 2 weeks. **(B)** Ratio of liver weight to body weight (n = 10–15 per group). **(C)** Liver triglyceride (TG) content was determined in liver extracts of *ob/ob* and *ob/ob*;*Shp* ^−/−^ mice (n = 4–5 per group). **(D, E)** Serum TG and free fatty acid (FFAs) contents under fasting conditions (n = 10–12 and n = 4–5 per group, respectively). Data are mean ± SEM. *P < 0.01, **P < 0.05, for differences within each genotype between control and troglitazone-treated mice. #P < 0.01, ##P < 0.05 for differences between control-treated *ob/ob*;*Shp* ^−/−^ and control-treated *ob/ob* mice.

Since SHP is normally highly expressed in the liver, we further investigated the potential effects of SHP deficiency in the *ob/ob* fatty liver. While the body weight showed no significant difference between *ob/ob* and *ob/ob*;*Shp*^−/−^ mice, the liver weight of *ob/ob*;*Shp*^−/−^ mice was significantly lower than that of *ob/ob* mice, resulting in a smaller liver/body weight ratio (9-10-week-old *ob/ob*;*Shp*^−/−^ liver weight 1.67 ± 0.17 g versus *ob/ob* 2.12 ± 0.18 g, P < 0.05) (Figure 2B). Histological analysis of the liver showed that lipid droplets were much smaller and less numerous in *ob/ob*;*Shp*^−/−^ mice than that in *ob/ob* mice, indicating an improvement of fatty liver in *ob/ob*;*Shp*^−/−^ mice (Figure 2A), and this was confirmed by measuring hepatic triglycerides (Figure 2C). These results are consistent with those described previously [36]. Troglitazone treatment of *ob/ob* mice caused a significant increase in liver/body weight ratio and hepatic triglyceride content (Figure 2B and C). Histological results also revealed that the size and number of lipid droplets were increased by troglitazone treatment in *ob/ob* mice (Figure 2A). However, these effects of troglitazone were not observed in *ob/ob*;*Shp*^−/−^ mice. In addition, the serum triglyceride- and FFA-lowering actions of troglitazone observed in the *ob/ob* mice were absent in *ob/ob*;*Shp*^−/−^ mice (Figure 2D and E). These results confirm that SHP is involved in the development of fatty liver in *ob/ob* mice and is required for hypolipidemic effects of TZDs.

### SHP deficiency downregulates the expression of lipogenic genes in *ob/ob* mice liver

**Figure 3:**
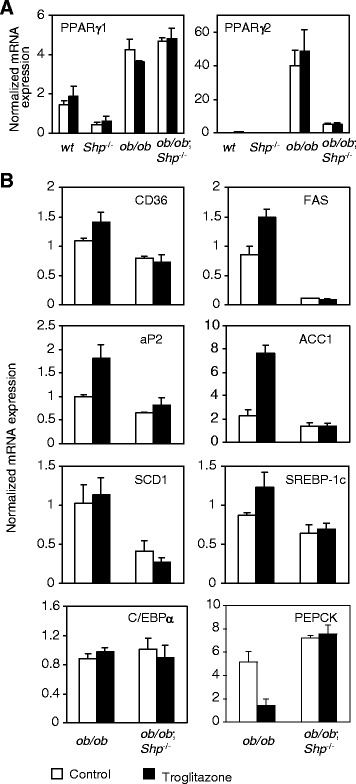
Expression of lipogenic genes was downregulated by SHP deficiency in *ob/ob* mice liver. Results in panels **A** and **B** are liver mRNA levels for control (open bars) and troglitazone-treated (filled bars) mice after 2 weeks of treatment. Data are expressed as relative fold change after normalized to 36B4 and are mean ± SEM (n = 4–5 per group). By two-way ANOVA, the genotype effect (*ob/ob* and *ob/ob*;*Shp* ^−/−^) is significant (P < 0.05) for all except PPARγ1 in panel **A**. The treatment effect and genotype × treatment interaction is significant for CD36, aP2, FAS, ACC1 and PEPCK in panel **B**.

Since hepatic PPARγ has been reported to play a critical role in the development of fatty liver of *ob/ob* mice [18], PPARγ1 and γ2 expression was examined in *wt*, *Shp*^−/−^, *ob/ob* and *ob/ob*;*Shp*^−/−^ mice (Figure 3A). The low basal PPARγ1 levels showed about a 3-fold increase in both *ob/ob* and *ob/ob*;*Shp*^−/−^ mice compared to *wt* mice, but PPARγ2 levels exhibited dramatic differences between *ob/ob* and *ob/ob*;*Shp*^−/−^ mice: a 40-fold increase in *ob/ob* mice relative to wild type, but only a 5-fold increase in *ob/ob*;*Shp*^−/−^ mice.

To further define the genes regulated by SHP in the *ob/ob* liver and the mechanism of the decrease in hepatic triglyceride in *ob/ob*;*Shp*^−/−^ mice, mRNA from livers of control- and troglitazone-treated mice was analyzed (Figure 3B). mRNA levels of fatty acid translocase (CD36), fatty acid synthase (FAS), adipocyte fatty acid-binding protein (aP2), acetyl-CoA carboxylase 1 (ACC1) and stearoyl-CoA desaturase-1 (SCD-1) were lower in *ob/ob*;*Shp*^−/−^ mice than in *ob/ob* mice. Troglitazone treatment induced the expression of CD36, FAS, aP2 and ACC1 mRNA in *ob/ob* mice, but not in *ob/ob*;*Shp*^−/−^ mice. There was no difference between *ob/ob* and *ob/ob*;*Shp*^−/−^ mice for expression of transcription factors regulating the lipogenic genes, such as SREBP-1c and C/EBPα . The mRNA levels of genes associated with glucose homeostasis, such as phosphoenolpyruvate careboxykinase (PEPCK) for gluconeogenesis, were increased in *ob/ob*;*Shp*^−/−^ mice (Figure 3B), which may partly account for the high blood glucose levels in these double mutant mice (Figure 1A). The action of troglitazone to lower glucose levels in type 2 diabetics by decreasing gluconeogenesis in liver was observed in *ob/ob* mice, but not in *ob/ob*;*Shp*^−/−^ mice.

In summary, expression of lipogenic genes was decreased by SHP deficiency in *ob/ob* mice, which has also been observed in Western diet fed *Shp*^*−/−*^ mice [39]. Consistent with the low expression of hepatic PPARγ2 in *ob/ob*;*Shp*^−/−^ mice, functional response to PPARγ agonist, troglitazone, was impaired in *ob/ob*;*Shp*^−/−^ mice both in lipogenesis and gluconeogenesis.

### SHP deficiency affects TZD-responsive gene expression in adipose tissue of *ob/ob* mice

**Figure 4:**
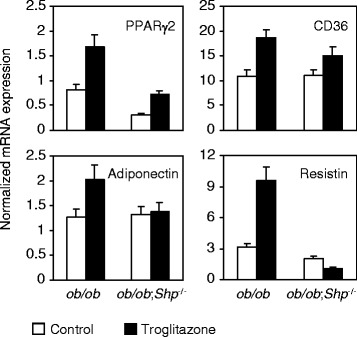
SHP deficiency affects TZD-responsive gene expression in adipose tissue of *ob/ob* mice. Results are adipose mRNA levels for control (open bars) and troglitazone-treated (filled bars) mice after 2 weeks of treatment. Data are expressed as relative fold change after normalized to 36B4 and are mean ± SEM (n = 4–5 per group). By two-way ANOVA, the genotype and treatment effect is significant (P < 0.05) for PPARγ2 and resistin (treatment effect for CD36, P = 0.06). The genotype × treatment interaction is significant only for resistin. By two-tailed *t* test, P < 0.05 for differences in adiponectin expression between control and troglitazone-treated *ob/ob* mice.

White adipose tissue has been thought to be the major site of TZD actions, as it is the only insulin-responsive tissue with high expression of PPARγ compared to liver and muscle [18]. Therefore, mRNA levels of genes responsive to TZDs in adipose tissue of control- and troglitazone-treated *ob/ob* and *ob/ob*;*Shp*^−/−^ mice were analyzed by real-time quantitative PCR analysis (Figure 4). Adipose tissue from *ob/ob*;*Shp*^−/−^ mice showed an approximately 60% reduction in PPARγ2 expression, which was not as dramatic as the nearly 90% reduction in the liver (Figure 3A). CD36 and adiponectin expression was not different between genotypes, whereas resistin decreased 35% in *ob/ob*;*Shp*^−/−^ mice. Troglitazone induced the expression of PPARγ2 and CD36 to a lesser extent in *ob/ob*;*Shp*^−/−^ mice than that in *ob/ob* mice, and failed to induce expression of adiponectin and resistin in *ob/ob*;*Shp*^−/−^ mice, demonstrating that SHP is required for full troglitazone responsiveness in adipose tissue.

### SHP upregulates PPARγ2 expression in primary hepatocytes

**Figure 5:**
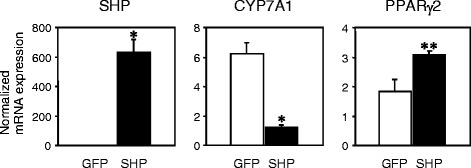
SHP increases PPARγ2 expression in primary hepatocytes. Expression of SHP, CYP7A1 and PPARγ2 in adenovirus-mediated GFP- or SHP-overexpressing hepatocytes by real-time quantitative PCR analysis. Hepatocytes were infected with green fluorescent protein (GFP) or SHP adenovirus as indicated. Data are expressed as relative fold change after normalized to 36B4 and are mean ± SEM (n = 4–5 per group). *P < 0.001, **P < 0.05, compared with GFP-infected cells.

To test the possibility that SHP might regulate PPARγ2 gene expression, the effects of SHP on the PPARγ2 gene were examined by infecting mouse primary hepatocytes with adenoviral vectors expressing SHP (Figure 5). Transduction of cultured hepatocytes with SHP adenovirus decreased expression of CYP 7A1 mRNA, a known SHP target gene, by 5.2 fold, while increasing PPARγ2 levels for 1.7 fold. These data indicate that SHP overexpression upregulates PPARγ2 expression in primary hepatocytes.

## Discussion

The therapeutic use of TZD drugs in the treatment of insulin resistance and type 2 diabetes is now well established [3-5]. TZDs act by increasing insulin sensitivity. These drugs are high affinity ligands for the nuclear receptor PPARγ [9,10] and their antidiabetic effects are thought to be mediated through PPARγ. Therefore, normal PPARγ expression levels, especially in the insulin-responsive tissues, are critical for TZD actions. In this study, we found that SHP deficiency causes downregulation of PPARγ2 expression in liver and adipose tissue of *ob/ob* mice, and these animals show diminished or abolished responsiveness to the TZD troglitazone. It is reported that mutations in SHP gene in humans are associated with insulin resistance and mild obesity [27]. Our data suggest that the diabetic syndromes of subjects with genetic mutations of SHP may not be improved by TZD treatment. This is the first *in vivo* evidence that SHP mutation attenuates TZD actions, which makes SHP a possible pharmacogenetic determinant of TZD response.

The *ob/ob*;*Shp*^−/−^ double mutant mice showed higher blood glucose levels than *ob/ob* mice. This may be attributed to abnormal glucose homeostasis in two tissues: skeletal muscle and liver, which have the greatest direct impact on plasma glucose levels. Whereas no difference was observed between *ob/ob* and *ob/ob*;*Shp*^−/−^ mice in gene expression of glucose oxidation and glycogen synthesis in muscle (data not shown), hepatic PEPCK mRNA expression is enhanced in *ob/ob*;*Shp*^−/−^ mice compared to *ob/ob* mice, suggesting that increased gluconeogenesis may contribute to elevated glucose levels. Moreover, the low insulin levels of *ob/ob*;*Shp*^−/−^ mice may indicate that pancreatic β cells fail to appropriately compensate for insulin resistance by increasing insulin secretion. It has been shown that independent of PPARγ activation, SHP positively regulates glucose-stimulated insulin secretion (GSIS) in β cells [40], which might be impaired in *ob/ob*;*Shp*^−/−^ mice.

The observation that *ob/ob*;*Shp*^−/−^ mice exhibit worse hyperglycemia and glucose intolerance than *ob/ob* mice contrasts with our previous report [36]. In that study, glucose and insulin levels were comparable in both genotypes and loss of SHP was associated with improved insulin sensitivity. One possible explanation is that the age and gender of the mice in the two reports are not exactly the same. The current studies focused solely on male mice at the age of 10 weeks, or older after 2 weeks of control or troglitazone treatment, whereas the prior study used age- and sex-matched groups of younger 8 week old mice. The age of the *ob/ob* mice may be particularly important since blood glucose rises in this time period before reaching a peak during 3–5 months of age. Thus, it is possible that earlier beneficial effects of the loss of SHP are not evident in these somewhat older mice. Consistent with this, we have observed very similar negative effects of the loss of SHP in long term studies of wild type and *Shp*^−/−^ mice fed a Western diet [39].

The loss of TZD responsiveness in the *ob/ob*;*Shp*^−/−^ mice is presumably a consequence of decreased PPARγ expression. PPARγ1 expression in adipose tissue, liver and muscle is not changed between genotypes, and PPARγ2 expression in muscle is unaffected (Figure 3A and data not shown). Thus, we conclude that loss of SHP primarily affects PPARγ2 expression, which is the dominant isoform in both adipose tissue and fatty liver [41]. Although the effect on PPARγ2 expression was strongest in the liver, a major SHP expressing tissue, decreased hepatic PPARγ2 cannot account for the loss of antidiabetic effects of TZDs, since rosiglitazone improved glucose homeostasis in liver-specific PPARγ knockout mice in the *ob/ob* background [18]. Although SHP is expressed at only low levels in adipose tissue [33,38], white adipose is the primary target of TZD actions and PPARγ2 expression was significantly decreased in *ob/ob*;*Shp*^−/−^ adipose tissue. Consistent with this, the induction of CD36 by troglitazone was decreased, and the response of both adiponectin and resistin was lost in the *ob/ob*;*Shp*^−/−^ adipose tissue. Adiponectin promotes fatty acid oxidation and insulin sensitivity in muscle and liver, and the antidiabetic effects of a low dose of pioglitazone were lost in mice in which adiponectin deficiency was introduced into the *ob/ob* background [42]. Thus, the absence of its induction is likely a major factor in the attenuated effects of troglitazone in the *ob/ob*;*Shp*^−/−^ mice. In contrast, elevated resistin has been proposed to increase insulin resistance, and the repression of resistin expression in normal mice by PPARγ agonists is thought to enhance insulin sensitivity [43,44]. Thus, the inductive effect of resistin by troglitazone in the *ob/ob* mice and the loss of this response in the *ob/ob*;*Shp*^−/−^ mice seems inconsistent with the known resistin action. Similar inductive effects of resistin by PPARγ activation have previously been described in both *ob/ob* mice and Zucker diabetic fatty rats [45], but this was not observed in another study [42]. The basis for these discrepant mRNA expression results, and also a substantial disconnect between resistin adipose mRNA and serum protein levels in *ob/ob* mice [46], remains unresolved.

SHP is involved in the development of fatty liver by regulating hepatic PPARγ2 and lipogenic genes. The increase in PPARγ2 appears to be a general property of steatotic liver in diet-induced and genetic obese models [18-20,47,48]. Overexpression of PPARγ in a hepatic cell line leads to marked lipid accumulation [49], as does overexpression in PPARα null livers [50]. Thus, SHP deficiency reduced the elevated levels of PPARγ2 and lipogenic genes in *ob/ob* liver, resulting in the improvement of fatty liver. Additional effects on other pathways may also contribute to the decreased triglyceride accumulation [36].

Recent studies have started to shed light on the molecular basis for the transcriptional regulation of PPARγ by SHP. Renga et al. described that FXR binds to the PPARγ promoter and activates its transcription via the recruitment of SHP in hepatic stellate cells [51]. Whereas Kim et al. identified a novel transcriptional cascade linking RAR/SHP signals with PPARγ2 expression through hairy and enhancer of split 6 (Hes6) in hepatocytes [52]. This transcriptional regulatory pathway controls hepatic lipid metabolism and provides a potential therapeutic entry point for NAFLD. These studies indicate that SHP appears to upregulate PPARγ expression by diverse mechanisms in different cell types.

## Conclusions

In summary, our results demonstrate that the antidiabetic and hypolipidemic actions of TZDs require the presence of SHP, likely due to the downregulation of PPARγ2 expression in the adipose tissue and liver of *ob/ob*;*Shp*^−/−^ mice. Thus, genetic or pharmacologic modulation of SHP activity could alter the efficacy of TZD antidiabetic actions.

## Abbreviations

ACC1: Acetyl-CoA carboxylase
aP2: Adipocyte fatty acid-binding protein
CD36: Fatty acid translocase
C/EBPα: CCAAT/enhancer binding protein alpha
FAS: Fatty acid synthase
FXR: Farnesoid X receptor
GFP: Green fluorescent protein
FFA: Free fatty acid
NAFLD: Non-alcoholic fatty liver disease
NASH: Non-alcoholic steatohepatitis
PEPCK: Phosphoenolpyruvate careboxykinase
PPARγ: Peroxisome proliferator-activated receptor gamma
RAR: Retinoic acid receptor
SCD-1: Stearoyl-CoA desaturase-1
SHP: Small heterodimer partner
SREBP-1c: Sterol regulatory element-binding protein 1c
TG: Triglyceride
TZDs: Thiazolidinediones
WT: Wild type
